# Invasion of the thoracic duct by postlaryngectomy stomal recurrence: a case report

**DOI:** 10.1186/s13256-020-02400-1

**Published:** 2020-06-13

**Authors:** Weiyu Zhu, Xinming Yang, Minghui Wei, Shuang Wang

**Affiliations:** 1 Department of Head and Neck Surgery, National Cancer Center/National Clinical Research Center for Cancer/Cancer Hospital & Shenzhen Hospital, Chinese Academy of Medical Sciences and Peking Union Medical College, Shenzhen, 518116 Guangdong PR China; 2 Department of Otolaryngology-Head and Neck Surgery, The Second Xiangya Hospital, Central South University, 139 Renmin Road, Changsha, 410011 Hunan PR China

**Keywords:** Postlaryngectomy stomal recurrence, Thoracic duct, Invasion, Chylous fluid, Case report

## Abstract

**Background:**

Postlaryngectomy stomal recurrence can infiltrate the adjacent tissues of tracheal stoma. However, to the best of our knowledge, there is no report about postlaryngectomy stomal recurrence invading the thoracic duct with intra-mass accumulation of chylous fluid.

**Case presentation:**

Our patient was a 52-year-old Han man who presented with a cystic-solid mass on the left side of the tracheal stoma after total laryngectomy. A diagnosis of postlaryngectomy stomal recurrence was confirmed by fine-needle aspiration and surgical dissection of the parastomal mass.

**Conclusions:**

In the case of parastomal masses in a total laryngectomized patient, the rare differential diagnosis of postlaryngectomy stomal recurrence invading the thoracic duct with intra-mass accumulation of chylous fluid should be considered a possibility.

## Introduction

Postlaryngectomy stomal recurrence (PSR) is the diffuse infiltration of a tumor at the junction of trachea and skin after laryngectomy. The occurrence rate of PSR reported in the literature ranges between 5 and 25% [1, 2]. PSR is a rare but well-recognized severe complication after total laryngectomy with nearly 80% mortality rate in the first 24 months [3, 4]. PSR can infiltrate the adjacent structures of tracheal stoma, such as esophagus and common carotid artery [2]. No case, however, of PSR invading the thoracic duct with intra-mass accumulation of chylous fluid has been reported in the previous literature. We confirmed via fine-needle aspiration and surgical dissection of the parastomal mass that a patient in our department had such a disease. The purpose of this case report is to remind clinicians about this rare differential diagnosis of parastomal masses in a total laryngectomized patient.

## Case presentation

**Fig. 1:**
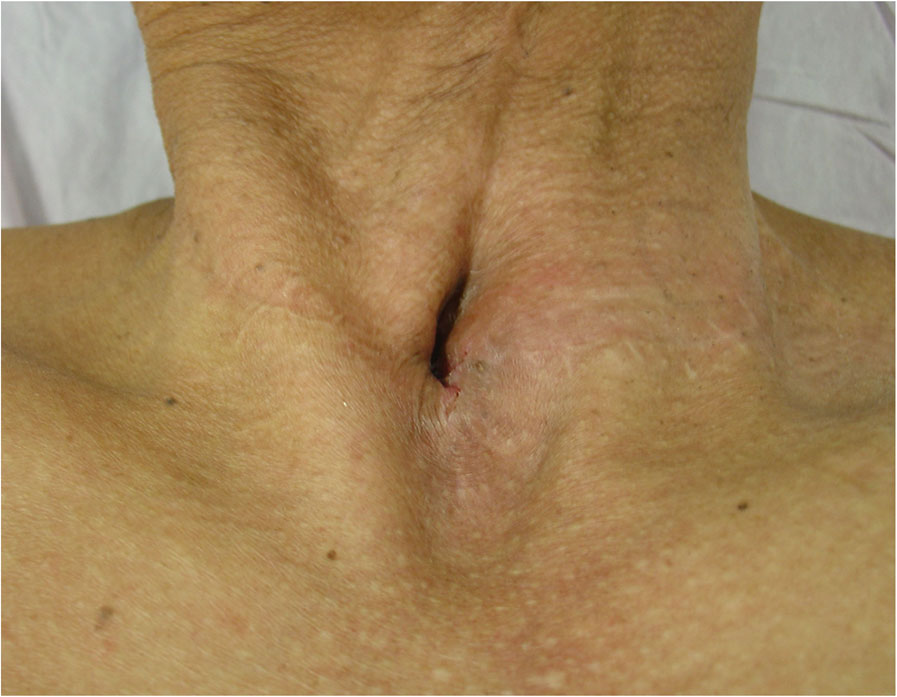
Preoperative image of a parastomal mass on the left side of the tracheal stoma. The mass extended the left lateral wall of the stoma and led to tracheostomal stenosis directly

**Fig. 2:**
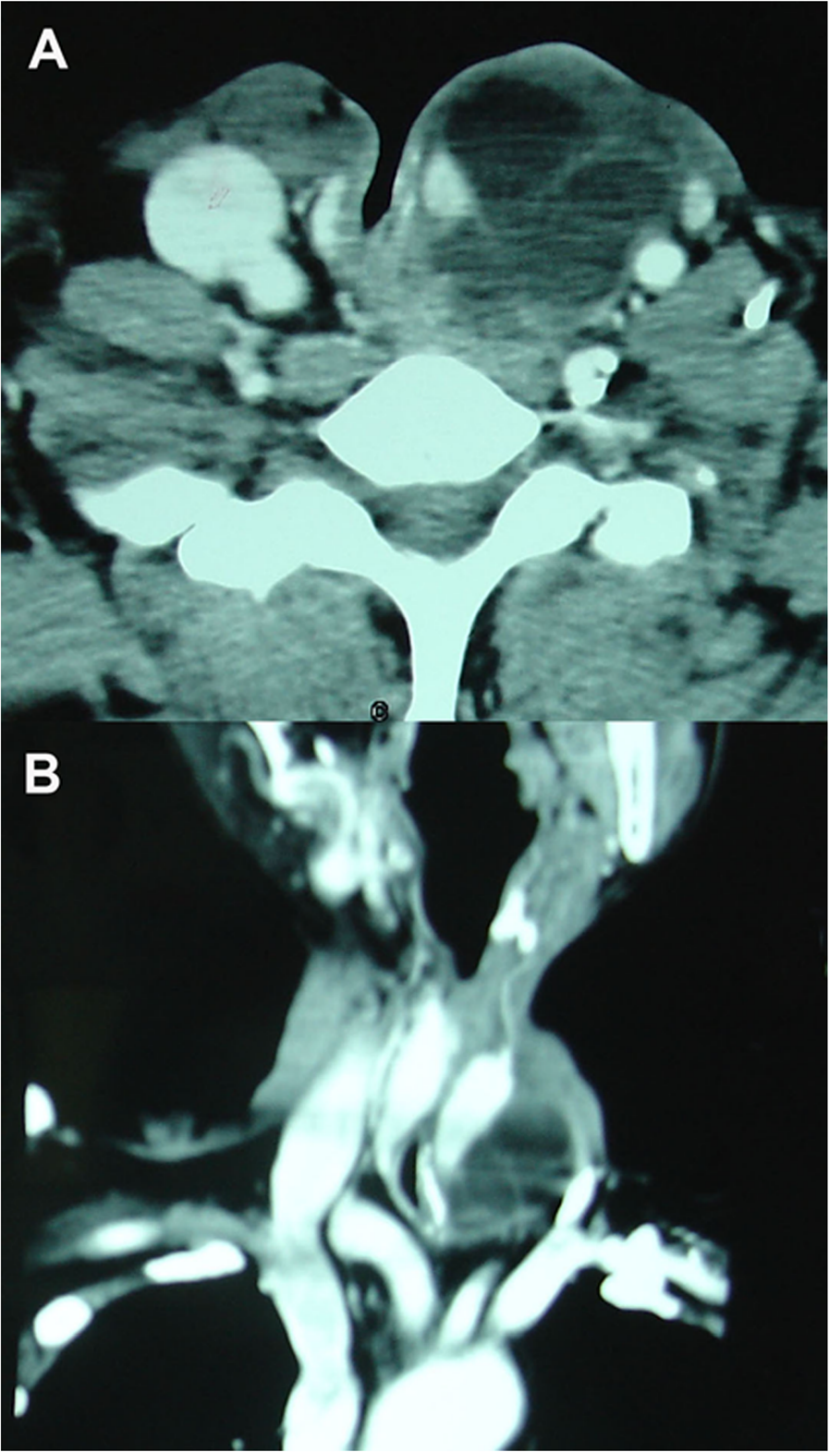
Axial (**a**) and coronal (**b**) computed tomography scans showed a well-circumscribed, non-enhancing, cystic-solid mass in the left side of the tracheal stoma

Our patient was a 52-year-old Han man who underwent partial laryngectomy in our department for squamous cell carcinoma of the glottis in September 2011. He came to our department again for recurrence of laryngeal carcinoma and underwent total laryngectomy and cervical lymph node dissection in November 2016. Then, he received radiotherapy at 60 Gy for 1.5 months. There was no obviously abnormal family history. He healed well until April 2018, when a slowly enlarging mass on the left side of the tracheal stoma was noticed. Since then, he had experienced progressively aggravating dyspnea. He came to our out-patient department in October 2018. A physical examination revealed an immobile, well-defined, partially compressible and non-pulsatile mass that measured approximately 4 cm × 4 cm on the left side of the tracheal stoma (Fig. 1). The mass extended from the left lateral wall of the stoma and led to tracheostomal stenosis directly. There was no lymph node enlargement in the remainder of his neck. Contrast-enhanced computed tomography of his neck revealed a well-circumscribed, non-enhancing, cystic-solid mass measuring 5 cm × 4 cm in the left side of the tracheal stoma (Fig. 2). A fine-needle aspiration of the mass yielded approximately 10 ml of yellowish-white fluid that contained a triglyceride level of 26.5 mmol/L, a protein level of 58.0 g/L, a glucose level of 8.5 mmol/L, and a white blood cell count of 1.0 × 10^9^/L, with 96% lymphocytes. The fluid was confirmed to be chyle on the basis of these results. The parastomal mass became smaller after the fine-needle aspiration, but returned to its original size on the following day. The same fluid was aspirated by fine-needle aspiration. Subsequently, he was admitted to our department. An ultrasonography of his neck described a 43 mm × 36 mm mixed echoic mass located between the left lobe of the thyroid gland and the great vessels. Other routine preoperative examinations were normal. According to his medical history, computed tomography, and the results of the fine-needle aspiration, a diagnosis of PSR invading the thoracic duct with intra-mass accumulation of chylous fluid was suspected.

**Fig. 3:**
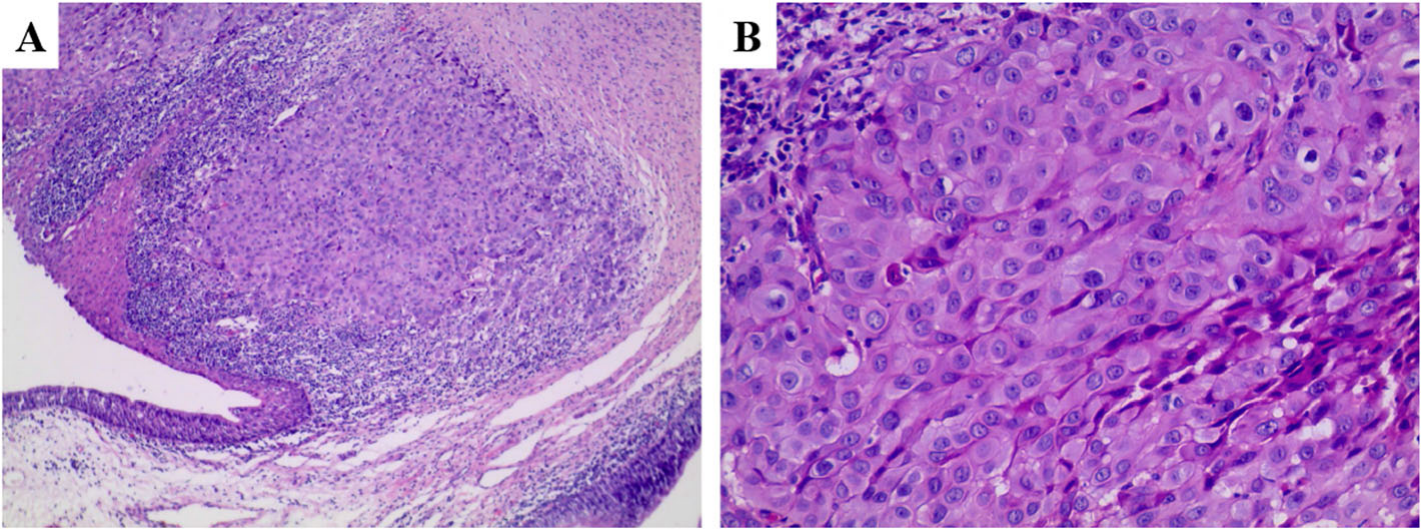
A hematoxylin and eosin stain of parastomal mass showed squamous cell carcinoma (original magnification **a**, × 10; **b**, × 40)

On October 29, 2018, a parastomal mass excision was performed through both cervical and thoracic incision. An intraoperative frozen section showed moderate differentiated squamous cell carcinoma (Fig. 3). The upper half of the manubrium sterni and the left sternoclavicular joint were resected to obtain a best exposure of the mass. Surgical dissection revealed that the lower part of the left internal jugular vein and the terminal segment of the thoracic duct were surrounded by the tumor. The thoracic duct was ligated and sutured by the edge of tumor approximately 1 cm and the left internal jugular vein was excised. Then, the mass was meticulously dissected free from surrounding tissues. The incision was closed following placement of one suction drain. Our patient developed chyle fistula after parastomal mass excision. Treatment with closed continuous vacuum drainage (− 400 mmHg) and total parenteral feeding resulted in the closure of the fistula. He has now been off surgery for 15 months and recent follow-ups have not revealed any evidence for tumor recurrence.

## Discussion

PSR is defined as the diffuse infiltration of recurrent cancer at the junction of the cervical skin and the amputated trachea as well as in the soft tissues adjacent to the tracheal stoma after total laryngectomy [1, 2]. Most cases of PSR are diagnosed within the first year after total laryngectomy and they die from the progression of the disease. Sisson *et al.* classified PSR into four types according to the location of tumor, which is correlate with the selection of appropriate treatment [5]. Type I is recurrent lesion located at the superior aspect of the stoma without esophageal involvement. Type II indicates that recurrent lesion is still located at the superior aspect of the stoma but with esophageal involvement. Type III originates from the inferior part of the stoma and usually has direct extension into the mediastinum. Type IV originates from the inferior aspect of both sides of the stoma and usually has a lateral extension beneath the clavicle. The 2-year survival rate for patients with Type I and II remains 45%, while the survival rate for Type III and IV was only 9% in 2 years [6, 7]. The major risk factors for PSR which have been reported are subglottic involvement, advanced tumor stage, preoperative tracheotomy, and paratracheal lymph node metastasis [8–10]. In our case, the classification of PSR can be attributed to Type I, and the risk factors for PSR are advanced tumor stage (T4N0M0) and preoperative tracheotomy.

After originating from the cisterna chyli, penetrating through the thorax, the thoracic duct enters the lower left side of the neck posterior to the common carotid artery and the internal jugular vein, and arches 3 to 4 cm above the clavicle. It lies anterior to the phrenic nerve, sympathetic trunk and thyrocervical trunk, and finally terminates into the venous system. The thoracic duct is generally supposed to terminate into the angle between the internal jugular and subclavian veins [11], but it may also terminate into the internal jugular, subclavian, external jugular or innominate veins [12].

Chyle has a very characteristic milky-white appearance and a very unusual composition in accordance with both biochemical and cytological analysis. The most notable among all results of biochemical analysis is the high concentration of triglycerides and chylomicrons. The results of cytological analysis will show lymphocytes to be the most predominant cell type, which may be another sign of chyle [13]. However, the value of proteins, electrolytes, and glucose in chyle is similar to those in plasma. Based on these characteristics of chyle, the fluid aspirated in our patient can be proven to be of chylous origin.

It is known that the tracheal stoma is located just above the suprasternal fossa which is close to the thoracic duct. The wall of the cervical thoracic duct, which is composed of a layer of connective tissue and smooth muscle fibers without any elastic lamina, is extremely thin [13]. Accordingly, for the reasons given above, the neoplastic tissue of PSR can easily invade and destroy the thoracic duct. The parastomal mass completely surrounds the terminal segment of the thoracic duct, which leads to the intra-mass accumulation of chylous fluid. That may explain the clinical manifestation of a slowly enlarging mass on the left side of the tracheal stoma in our case.

Diagnostic considerations for a parastomal, partially compressible cervical mass which manifests as a cystic-solid and non-enhancing mass lesion in a total laryngectomized patient, in our opinion, include PSR or enlarged lymph node accompanying central necrosis. However, a diagnosis of PSR invading the thoracic duct with intra-mass accumulation of chylous fluid may not be considered. Therefore, in order to better deal with the tumor and thoracic duct during the operation and reduce postoperative complications, this rare differential diagnosis of parastomal masses should be considered before operation. Fine-needle aspiration is essential for a mass of this type in a total laryngectomized patient because of its important role in preoperative diagnosis.

## Conclusion

In the case of parastomal masses in a total laryngectomized patient, the rare differential diagnosis of PSR invading the thoracic duct with intra-mass accumulation of chylous fluid should be considered a possibility.

## Data Availability

Not applicable.

## Abbreviation

PSR: Postlaryngectomy stomal recurrence
